# Supplementary material for: Comprehensive Analysis of Cuproptosis-Related Genes in Prognosis and Immune Infiltration of Hepatocellular Carcinoma Based on Bulk and Single-Cell RNA Sequencing Data
Source: Cancers (Basel). 2022 Nov 21;14(22):5713. doi: 10.3390/cancers14225713 (PMC9688556; doi:10.3390/cancers14225713)
Supplement: Supplementary file 1 [file cancers-14-05713-s001.zip › cancers-2056445-supplementary.pdf]

Supplementary Materials:

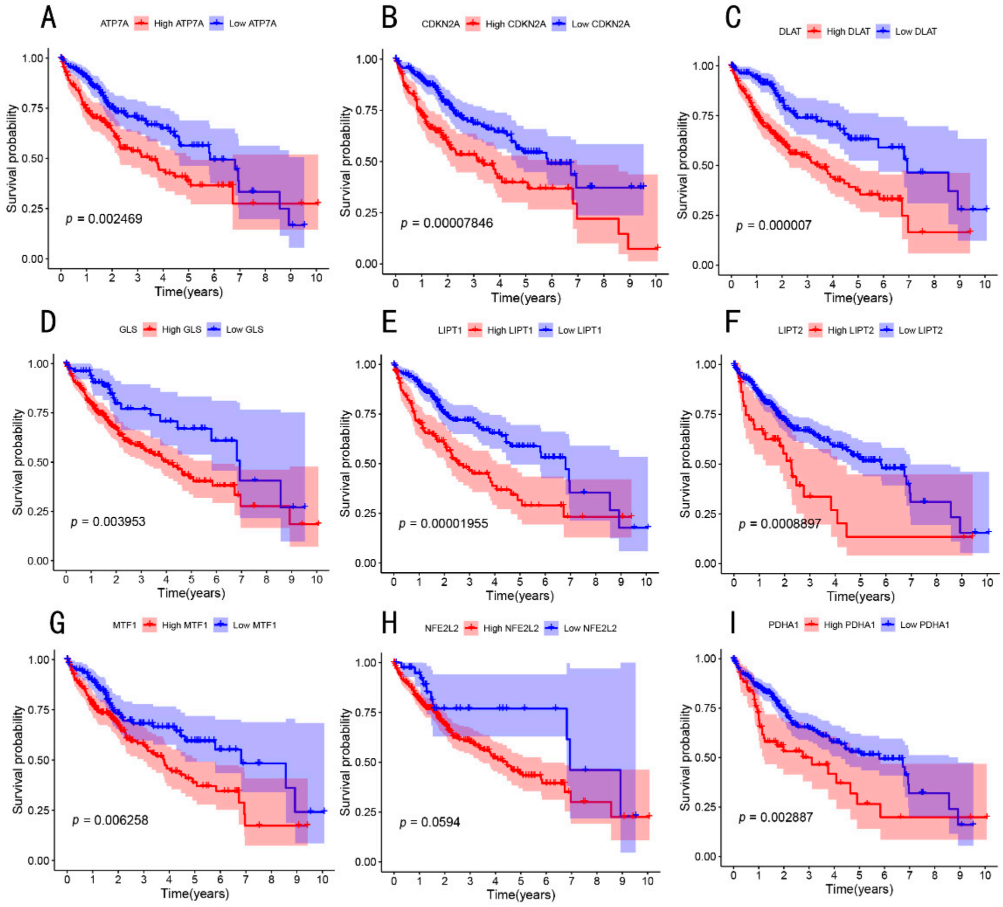

Figure S1. Survival analysis of nine cuproptosis-related prognostic genes ATP7A(A), CDKN2A(B), DLAT(C), GLS(D), LIPT1(E), LIPT2(F), MTF1(G), NFE2L2(H), PDHA1(I), obtained by univariate Cox regression analysis. The patients with high expression of ATP7A、CDKN2A、DLAT、GLS、LIPT1、LIPT2、MTF1 or PDHA1 had lower OS ($P < 0.05$).

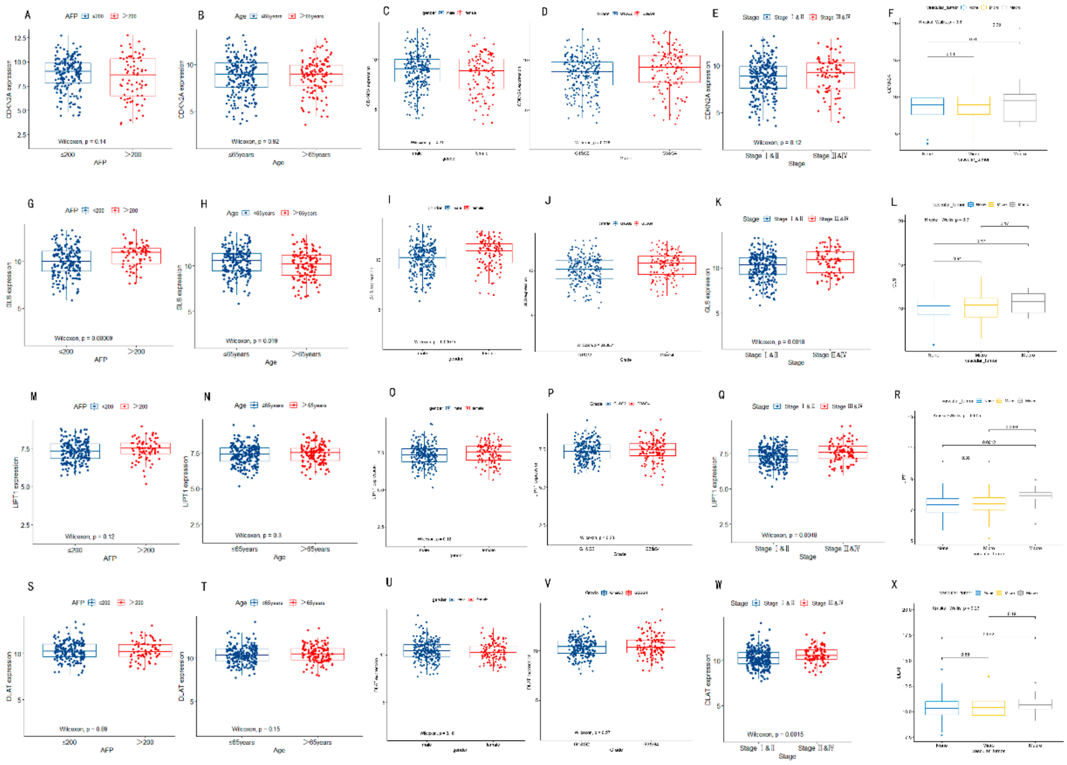

Figure S2. The relationship between clinicopathological characteristics and four genes in TCGA cohort. CDKN2A（A-F）、GLS（G-L）、LITP1（M-R）、DLAT（S-X）.

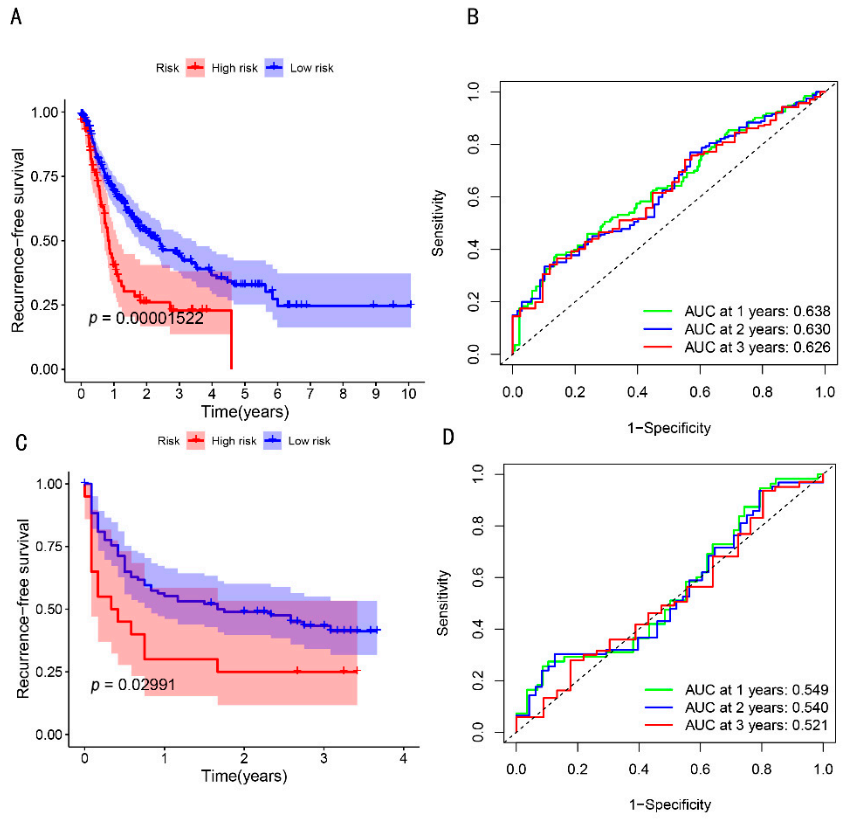

Figure S3. Four-gene signature model predicts recurrence-free survival in TCGA cohort (A) and in-house cohort data(C). Time-dependent ROC curves (B) and (D) of risk score prognostic performance.

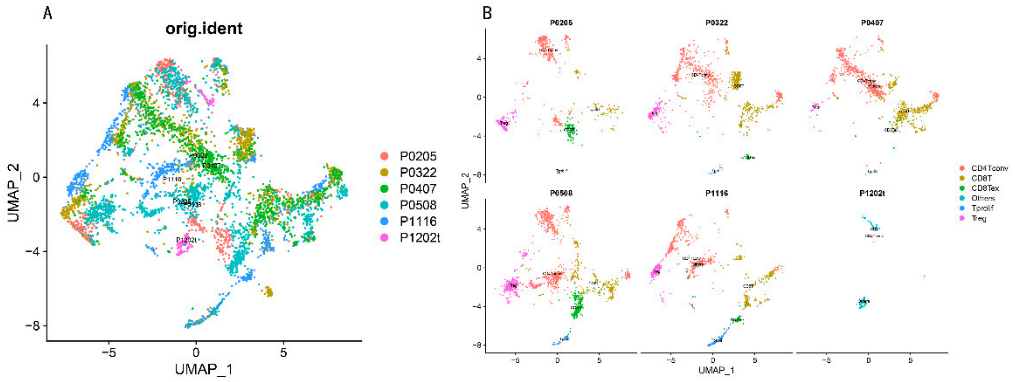

Figure S4. (A) UMAP plot showing immune cell distribution from 6 samples based on scRNA-seq. (B) UMAP plot showing the distribution of the six cell types of each sample for 6 samples
